# Level of knowledge and attitude of spanish primary school teachers regarding tooth avulsion

**DOI:** 10.4317/jced.62148

**Published:** 2024-10-01

**Authors:** Ana Martínez-Fuentes, Manuel Pabón-Carrasco, Isabel Crespo-Gallardo, Pablo Castelo-Baz, Juan José Segura-Egea, Jenifer Martín-González, Alberto Cabrera-Fernández

**Affiliations:** 1 Department of Stomatology, Endodontic Section, School of Dentistry, University of Sevilla, Sevilla, Spain; 2 Department of Nursing, Faculty of Nursing, Physiotherapy and Podiatry, University of Sevilla, Sevilla, Spain; 3 Oral Sciences Research Group, Endodontics and Restorative Dentistry Unit, School of Medicine and Dentistry, University of Santiago de Compostela, Health Research Institute of Santiago de Compostela (IDIS), 15706 Santiago de Compostela, Spain

## Abstract

**Background:**

Nowadays, traumatic dental injuries (TDIs) have a growing prevalence and incidence worldwide, reaching their highest numbers in school-age children. The management of dental avulsion requires an important challenge for the clinicians and the prognosis depends on an immediate and an appropriate emergency action. In this regard, primary school teachers are in a privileged position to assist injured children. Objective: To assess the knowledge and attitudes of primary school teachers regarding the proper management of dental avulsion in schoolchildren.

**Material and Methods:**

This cross-sectional study was conducted in Spain, including teachers from schools distributed throughout the Spanish territory, selected randomly. The sample consisted of 240 teachers. An ad hoc questionnaire, distributed in physical and online formats, was used to assess their knowledge on the proper management of dental avulsion in schoolchildren.

**Results:**

A very high response rate was obtained (80%). The majority of the participants were unable to reimplant the tooth (76.3%), and they lacked knowledge of the appropriate medium for its optimal preservation (85.8%).

**Conclusions:**

This study suggests that there is an inadequate level of knowledge about the management of dental avulsion injuries among primary school teachers in Spain. It would be necessary to design educational strategies to improve this situation.

** Key words:**Primary school teachers, traumatology, avulsion, knowledge.

## Introduction

Nowadays, traumatic dental injuries represent an emerging public health problem. These injuries involve the acute transmission of energy to the tooth and/or its supporting tissues, leading to a wide range of severe consequences (1).

The term “avulsion” describes the situation in which a tooth is completely displaced from the alveolar socket due to a severe trauma. This process is considered one of the most significant traumatic injuries, requiring immediate attention (2).

According to available scientific literature, traumatic dental injuries (TDIs) are very prevalent, accounting for 85% of oral injuries. These injuries represent 5% of bodily injuries, despite the oral region comprising only 1% of the entire body (3).

A recent meta-analysis indicated that the global prevalence of TDIs in permanent dentition was 15.2%. In Europe, the prevalence is 14% (4). The highest incidence of these dental events is observed between the ages of 7 and 12 . In this age group, the alveolar bone offers minimal resistance to extrusive forces, which predisposes to such injuries (3). Specifically, dental avulsion accounts for 0.5-6.2% of all traumatic accidents in permanent dentition. Upper central incisors are the most commonly affected teeth. Regarding gender, TDIs are more frequent in boys than in girls (4). Although the prevalence and incidence of TDIs are high worldwide, they can vary depending on socio-economic, behavioral, or cultural differences among different nations (4).

On the other hand, a study conducted in Spain by Faus-Damiá *et al*. estimated the incidence of TDIs in school-age children in 6.2% (5). Etiological factors are closely related to the patient’s age (6,7).

In respect of the management of dental avulsion in permanent dentition, it requires a therapeutic challenge, with the prognosis varying greatly depending on the actions taken at the time of the accident and immediately after. Therefore, treatment strategies for the affected tooth must be determined as soon as possible, with emergency care being crucial for tooth preservation (1,2).

Inadequate assistance for this trauma in children can affect growth, function and phonetics aspects, leading to an impairment in the child’s psychosocial development (8). So, to achieve an optimal prognosis for dental avulsion in terms of having a proper first-aid training , it’s essential not only for dental healthcare professionals but also for any person close to the child at the time of the accident, including parents (9).

Due to the significant amount of time children spend in schools daily, there is a high likelihood that school professionals, specifically teachers, are on the front lines of attending to children. However, various worldwide studies conducted to assess the knowledge of schoolteachers about dental avulsion and first aid have shown that their knowledge in this area is weak, and they rarely know how to respond to this critical situation (10–21). In our country, only one recent study evaluated the knowledge level of teachers in the province of Seville (22), but no survey has been conducted investigating this issue at the national level.

Therefore, the aim of this study was to evaluate the level of knowledge of spanish primary school teachers regarding the emergency management of dental avulsion, as well as to understand teachers’ self-perception regarding this issue and their willingness to acquire further knowledge.

## Material and Methods

-Study design

The present study is a cross-sectional study conducted in accordance with the STROBE guidelines for the presentation of cross-sectional studies (suppl. 1).

-Sample 

The study was conducted in Spain using random sampling with a 1:1 ratio sequence, employed to recruit primary school education professionals (PSEP). The schools were distributed through the spanish territory. A total of 260 participants were deemed, all of them signed an informed consent form.

The inclusion criteria applied were that participants should hold a recognized Bachelor’s or Diploma degree in Primary Education, as granted by the Spanish government. Furthermore, they should be actively practicing within the Spanish territory at the time of the questionnaire administration. Those individuals who, despite holding the required degree, either had never had contact with primary education schools or were not currently working in the field, were excluded.

In January 2023, surveys were distributed to the schools randomly selected from across the entire Spanish territory. The included schools were public, private, and semi-private (concerted) institutions, located in urban and rural areas. Additionally, schools of both secular and catholic identity were represented.

For distribution, a digital format was used, with surveys primarily sent via email. This method was also combined with physical delivery for those schools that requested it.

-Questionnaire

To conduct the study, a customized questionnaire was used, which collected sociodemographic variables as well as questions related to knowledge about different aspects about avulsion. This questionnaire had been previously used and validated in prior research (11).

The questionnaire exclusively comprised closed-ended questions and was structured into four sections. The first section gathered participants’ sociodemographic variables, including age, years of experience, and qualification level. The second section included three questions related to teacher training: first aid training, experience with dental avulsion, and prior information. A third section aimed to assess the level of knowledge, comprising six questions concerning the degree of urgency regarding referral, reimplantation, manipulation, and transportation. Finally, a fourth section sought to evaluate the attitudes of the PSEP towards further training and education regarding dental avulsion. The questionnaire was carried out in its spanish version.

The attitude section was categorized as positive or negative based on a “yes” or “no” response (Suppl. 2).

Once collected, six questions were deemed “evaluable,” and a score between 0 and 6 was assigned based on the number of correct responses.

-Statistical Analysis

The sample size was calculated to achieve a power of 0.95, with an alpha error of 0.05 and an effect size of 0.3 (test method: Chi-square test, G*Power 3.0.10, Franz Faul, University of Kiel, Kiel, Germany). A total of 220 participants were deemed. The sample size was increased by 15% to account for potential losses.

Descriptive analysis of qualitative variables was performed using frequencies and percentages for all variables. To assess the association between categorical variables, the Chi-square test of independence was used or, if this was not required, Fisher’s exact test. This test allows us to determine whether there is a significant relationship between two categorical variables. However, since a significant value of the Chi-square test does not provide information on the magnitude of the association, Cramer’s V coefficient was calculated to measure the effect size (0.1 indicates a small effect size, 0.3 indicates a medium effect size and 0.5 indicates a large effect size).All analyses were performed with SPSS® version 29.0. A significance threshold of *p* < 0.05 was adopted.

## Results

Of the total number of surveys distributed, 247 were finally answered and 7 were discarded because they were not properly filled in. The demographic data of the participants are collected in  Table 1.

Regarding previous experience in primary education, 13.6 ± 10.8 years of average experience was obtained among the respondents, with 45.4% of the respondents having less than 10 years of experience. Diploma was the most common level of education (44.6%) (107) among the participants. Ninety respondents (37.1%) were in current possession of a first aid certificate, and only 11 (4.6%) had received previous advice about dental avulsion. However, 42 respondents (17.5%) had ever witnessed a case of avulsion ( Table 1).

Among those who responded, 173 (72.1%) following a dental avulsion would first attempt to contact the parents or legal guardian before providing assistance. The majority of participants (183, 76.3%) were unable to reposition the avulsed tooth ( Table 2).

The questionnaire also asked about the management of an avulsed tooth contaminated and 118 respondents (49.2%) stated that they had no knowledge of how to proceed. To the same question, 84 (35.4%) would opt to rinse the tooth under running water for at least 10 seconds ( Table 2).

When the teachers were asked to evaluate the degree of confidence in their ability to handle this type of accident, 97.9% (235) stated that they did not have the necessary knowledge and an identical percentage indicated the need for further training ( Table 2).

**Figure 1:**
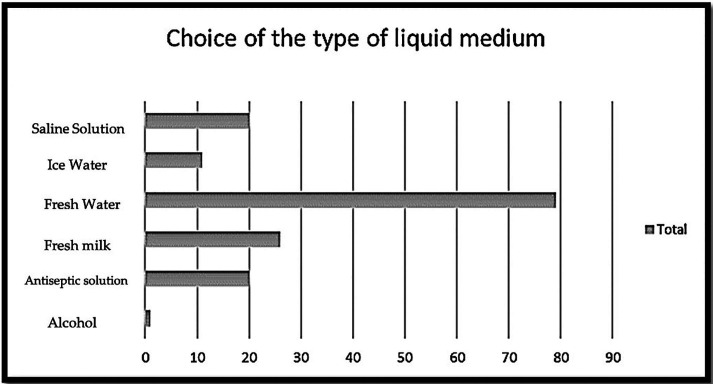
Answers regarding the most appropriate type of liuid medium to preserve the avulsed tooth.

When they were asked about the most appropriate technique for transporting the tooth, 63 respondents (26.7%) would keep the tooth in paper, and 63 (26.3%) considered it more appropriate to use a liquid medium. When they were asked which liquid medium they would use, 114 respondents (47.5%) opted for fresh water, and 42 (17.5%) chose saline solution (Fig. 1). A correlation was found with the variables sex, age range, experience, possession of a first aid certificate, previous experience of avulsion in dental traumatology and a higher level of knowledge as well as a proactive attitude ( Table 3).

Regarding the sex of the participants, a better choice of medium was observed in females with only 8.8% of participants opting for antiseptic solutions compared to 27.7% in males (*p*=0.013). Regarding the years of experience, a better attitude towards reimplantation of the avulsed tooth was observed the greater the professional experience, mainly in the 30-40 years of experience range with 34% intention to reimplant (*p*=0.036) ( Table 3).

With regard to the first aid certificate, it was found that those professionals who did not have a first aid certificate were less willing to reimplant (*p*=0.017) and had a worse feeling of training (*p*=0.050). Furthermore, a worse management of the avulsed tooth was evident (*p*=0.015).

Finally, with regard to previous avulsion, it was found that having previously witnessed a case of tooth avulsion had a significant influence on the variable “degree of urgency” (*p*=0.0001). It was also found that those who had witnessed avulsion in the past showed better judgement in choosing the correct type of liquid media (*p*=0.025) and a better attitude to reimplantation (*p*=0.014). Along the same lines, a weak or moderate association was observed for most of the variables.

## Discussion

This study aimed to evaluate the knowledge and attitudes of spanish primary school teachers regarding the emergency management of dental avulsion. The results show that most of spanish primary school teachers are unable to reimplant the avulsed tooth and they lacked knowledge of the appropriate medium for its optimal preservation. These findings show an inadequate level of knowledge about the management of dental avulsion injuries among primary school teachers in Spain.

The questionnaire used for this cross-sectional survey was validated in previous published studies (11). The form was distributed to primary education centres in different regions of Spain, with a high response rate (80.3%), significantly higher than the one reported by Berguer *et al*. (23) with only 47%. Thus, the sample can be considered representative of the spanish population of primary school teachers.

Antunes *et al*. (24) demonstrated that patients who had experienced dental trauma had a strong negative impact on their emotional well-being compared to those who had not. In addition, the high economic costs that may result from improper emergency intervention must be considered. To avoid all of this, it is crucial to assist the child by providing rapid and appropriate emergency care (6).

Regarding first aid training, only 37.1% of the teachers had received any course on it. This percentage is slightly lower than that found in similar studies(11,12).

When asked about avulsion and knowledge regarding its management, a vast majority (94.2%) declared having no knowledge. This percentage contrasts with the study by Zakirulla *et al*. (15), in which nearly 30% of the respondents had received training in dental traumatology and, therefore, in avulsion. Furthermore, this data becomes even more impactful when considering that 17.5% of the teachers reported having witnessed dental avulsion at some point. Other studies (16) report a Figure as high as 42.8%.

The prolonged dry storage of the avulsed tooth for more than 20-30 minutes results in the loss of normal physiological metabolism and the morphology of periodontal ligament (PDL) stem cells (2,25), which are essential for the reinsertion of the avulsed tooth and are considered to die after 60-120 minutes (2,6). However, Andreasen *et al*. (25) recommend reimplantation even after long extraoral periods. Therefore, it is crucial to refer the child with a dental traumatism for treatment as soon as possible. In the current study, 14.2% of the respondents believed that emergency treatment should be provided without necessarily contacting the parent or guardian, while 72.1% would attempt to contact the parents beforehand. These data are similar to those obtained in previous studies conducted in Australia, India, or Croatia (11,14,17).

Regarding the type of specialist, a high percentage (69.6%) chose the dentist as the ideal professional to assist the child. This reflects the high level of confidence teachers have in dentists to provide rapid and appropriate intervention, although in studies like Junges *et al*. (26), almost all teachers (94.5%) had a more clear view of this issue.

However, immediate reimplantation of the tooth (<15 minutes) at the accident place is much better. In this regard, participants were asked if they would be able to do the reposition of the tooth in its socket, with only 22.1% responding positively. While this percentage is quite modest, it is similar to that obtained by a previous study (11). More pessimistic results can be found in research carried out in the UK (20) or China (27), with data around 5%.

A more detailed analysis of the results revealed that teachers with more professional experience were up to 1.96 times more likely to be willing to reimplant the tooth compared to low professional experience (*p* < 0.05). This statistically significant difference could be explained by the greater likelihood that more experienced teachers have of having experienced similar situations in the past.

In any case, proper handling of the tooth after it has been removed from the socket is necessary. In addition, the tooth should always be held by the crown, without touching the root to avoid damaging the periodontal ligament cells (1). Furthermore, if the tooth has come into contacted with a dirty surface, it should be rinsed with cold running water or saline for 10 seconds (1,2), avoiding antiseptic solutions like soap. Only 35.1% of the participants selected the correct option and 49.4% claimed not to know how to proceed.

In cases where immediate reimplantation is not possible, certain conditions must be observed to ensure the survival of the periodontal ligament cells, making it essential to opt for a liquid medium (1). When spanish teachers were asked about their preferred transportation method, 39.2% opted for dry mediums. These data are similar to those reported in other international research, such as the study by Olatosi *et al*. (16), but more hopeful than those found in the study by Salaric *et al*. (28).

The literature presents a wide variety of mediums commonly used for transporting avulsed teeth. One of them is Hank’s Balanced Salt Solution (HBSS), which some authors have pointed to as the most suiTable medium due to its ability to maintain periodontal vitality for extended periods without the need for refrigeration. However, its limited accessibility meant it was not one of the options in our questionnaire (29). One of the most well-known mediums is water. However, its high hypotonicity can cause irreversible damage to periodontal ligament cells. Water should only be used in cases where no other alternative is available (29). More than half of the teachers (54.5%) chose water (in liquid or solid form) as their transportation medium. This percentage is higher than the results of most other published studies (10–21). Saline solution has been evaluated in various publications, revealing that storing the tooth in this medium causes cell membrane lysis, leading to its disuse (29). Our results showed that 17% of respondents selected this medium as the most suiTable, data similar to those were found in other countries like India (12). Saliva is one of the commonly suggested options, either in the child’s mouth or an adult’s mouth. There is a high risk of cell membrane infection and ingestion, leads us to discourage its use(29). Only 7.9% of our sample chose saliva, which could be attributed to awareness of the high risk of accidental ingestion. Finally, milk is characterized by its physiological properties regarding to the viability of periodontal ligament cells. This, along with its easy availability, lead to consider it an ideal medium, being referred to as the “Gold Standard” by the International Association of Dental Traumatology. However, refrigeration is important, and there are also studies that suggest skim milk may be more suitable (29). Despite this, only 14.2% of the teachers indicated that they would use it to transport the tooth. These results contrast with those collected from Australian teachers by Khan *et al*. (14), who seem to have greater knowledge in this area (49.5%). On the other hand, studies like the one conducted in Colombia (10) reflected a more pessimistic situation (6.5%).

In this research, when analyzing whether possession of a first aid certificate influenced the proper handling of avulsed teeth, statistically significant differences were observed (*p*=0.015). Teachers trained in first aid were up to 1.93 times more likely to correctly manage avulsed teeth following an accident. Knowledge of emergencies also proved to be influential in the willingness to reimplant the tooth and the positive predisposition to further education. These results highlight that healthcare training, even when unrelated to oral topics, has a positive and influential impact on adopting more appropriate attitudes for managing emergency situations. Therefore, it should be promoted among teachers.

The final part of our survey aimed to assess the satisfaction of primary school teachers with their level of training in this area and their willingness to improve their knowledge. A resounding 97.5% of the participants indicated that they were not satisfied with their knowledge of avulsion and were opened to participating in strategies to enhance their ability for such emergencies. These findings align with the trends in most countries, with very similar data reported in almost all available studies (10–21). Based on the results presented throughout the study, it is evident that there is a need to improve the training of spanish teachers in dental avulsion first aid. The available literature provides various strategies to achieve this, but there is no ideal method since there are no studies comparing the effectiveness of all educational strategies in this field. A significant controversy with opposing views is encountered, but all available studies appear to concur on the importance of active learning (30). Even more, it is worth noting that the majority of teacher education programs in spanish universities do not include subjects promoting oral health or addressing the most common dental emergencies in classrooms. Feldens *et al*. (19) suggested incorporating the management of traumatic dental injuries into the curriculum and pedagogical education of teachers.

This study has some limitations. In one hand, it has inherent limitations related to the chosen design. Cross-sectional descriptive studies cannot establish causality. On the other hand, the small sample size reduces the external validity of the findings. It is acknowledged that in some variables, there might be a risk of committing a type II error due to the small sample size. Nevertheless, significant gaps in teachers’ knowledge of dental trauma are observed, which hinder immediate assistance and put the viability of the dental piece at risk.

## Conclusions

Primary school teachers in Spain have a low knowledge of the initial management of a dental avulsion emergency. In addition, a lack of first aid training in dental trauma was observed. In addition, a positive predisposition to change the current situation is evident, being open to receive specific training to enhances the management of such events.

The present study highlights the need for promotion and educational policies for primary school teachers with the consequent community benefit that this entails.

## Figures and Tables

**Table 1:** Socio-demographic variables and previous education of participants.

Socio-demographic variables		
		n (%)
Sex	Men	47(19.6)
	Women	193(80.4)
Age	Young adult 20-24	17(7.1)
	Middle age 25-44	156 (65.0)
	Advanced age 45-64	67 (27.9)
Level of education	PhD	5 (2.1)
	Master	29 (12.1)
	Expert	2 (0.8)
	Lincensing	46 (19.2)
	Grade	51 (21.3)
	Diplomate	108 (44.6)
Professional experience	1 - 10 years	109 (45.4)
	10 – 20 years	74 (30.8)
	20 – 30 years	34 (14.2)
	30 – 40 years	23 (9.6)
First aid certificate	Yes	89 (37.1)
	No	141 (58.8)
	Don't know	10 (4.2)
Previous training		
Previous avulsion training	Yes	11 (4.6)
	No	226 (94.2)
	Don´t know	3 (1.3)
Previous avulsion experience	Yes	42 (17.5)
	No	188 (77.9)
	Don't know	11 (4.6)

Note: n=Frequency; %=Percent

**Table 2:** Knowledge and attitudes towards dental avulsion.

	n (%)
Knowledge	
Referral emergencies	
Very urgent, ask for help immediately without consulting parents	34 (14.2)
Try to inform the parents and then ask for help immediately, even if there is no response from the parents	173 (72.1)
Wait until the end of the day and inform parents at the end of the school day	19(7.9)
Doesn't know	14(5.8)
Management of the avulsed dirty tooth	
I would reattach the avulsed tooth without cleaning it	3(1.3)
I would use the child's saliva to wash the tooth out of the mouth and then put it back	16(6.7)
I would rinse the tooth under water for at least 10 seconds	84 (35.1)
I would wash the tooth with milk	14 (5.9)
I would wash the tooth with soap	0 (0.0)
I would rub the tooth gently with a toothbrush	4 (1.7)
Doesn't know	118(49.4)
Transport technique	
Liquid medium	63(26.3)
Ice	14(5.8)
Child's mouth	18(7.5)
Adult's mouth	1(0.4)
Child's hand	0(0.0)
Wrapped in paper	64(26.7)
Plastic packaging	30(12.5)
Don't know	50(20.8)
Specialist to refer	
Pediatrician	20(8.3)
Family doctor	8(3.3)
Maxylofacial	45(18.8)
Dentistry	167(69.6)
Attitudes	
Would you be able to implant an avulsed tooth?	
Yes	53(22.1)
No	183 (76.3)
Don't know	4(1.7)
Do you feel that you have sufficient knowledge to manage a dental avulsion injury among your students?	
Yes	6(2.5)
No	234(97.5)
Do you think you would need more knowledge/training on dental avulsion?	
Yes	235(97.9)
No	5(2.1)

Note: n=Frequency; %=Percent

**Table 3:** Inferential analysis of the socio-demographic variables with respect to the knowledge and attitudes of the participants.

Knowledge						Attitudes		
	Referral emergencies	Management of the avulsed dirty tooth	Transport technique	Choice of liquid medium	Specialist to refer	Training needs	Attitude to Re-implantation	Prior perception of knowledge
Sex								
X^2^	1.357	6.995	12.149	16.168	0.475	0.001	1.914	3.615
p value	0.715	0.321	0.059	0.013*	0.967	0.981	0.384	0.057
Cramer's V	0.070	0.170	0.220	0.300	0.040	0.020	0.080	0.120
Age ranges							
X^2^	3.839	1.357	10.642	19.869	6.776	0.648	5.636	1.724
p value	0.698	0.436	0.560	0.070	0.342	0.723	0.228	0.422
Cramer's V	0.08	0.150	0.140	0.20	0.110	0.050	0.100	0.080
Years of professional experience								
X^2^	5.833	17.657	17.502	17.455	12.068	6.519	X13.458	X2.203
p value	0.756	0.478	0.489	0.493	0.210	0.089	0.036*	0.531
Cramer's V	0.09	0.150	0.150	0.150	0.120	0.160	0.356	0.090
First aid certificate								
X^2^	4.829	4.829	19.803	10.943	6.174	3.390	12.086	5.661
p value	0.566	0.015*	0.071	0.534	0.404	0.184	0.017*	0.050^*^
Cramer's V	0.102	0.355	0.200	0.154	0.110	0.115	0.250	0.208
Previous avulsion experience								
X^2^	39.240	13.160	16.077	23.356	9.418	1.447	12.437	0.308
p value	0.001***	0.358	0.188	0.025*	0.151	0.485	0.014**	0.857
Cramer's V	0.415	0.165	0.181	0.207	0.140	0.080	0.262	0.032

Note: X2=Chi-square test, Cramer’s V=0.1 indicates a small effect size, 0.3 indicates a medium effect size and 0.5 indicates a large effect size; Significance set at *p*< 0.05*. *p*< 0.01**; *p*< 0.001***.

## Data Availability

The datasets used and/or analyzed during the current study are available from the corresponding author.
